# Identification of 4 immune cells and a 5-lncRNA risk signature with prognosis for early-stage lung adenocarcinoma

**DOI:** 10.1186/s12967-021-02800-x

**Published:** 2021-03-26

**Authors:** Lan Mu, Ke Ding, Ranran Tu, Wei Yang

**Affiliations:** 1 Department of Thyroid Surgery, The Second Xiangya Hospital, Central South University, Changsha, 410000 Hunan China; 2 Department of Urology, Xiangya Hospital, Central South University, Changsha, 410000 Hunan China; 3 Department of Neurology, The Second Xiangya Hospital, Central South University, Changsha, 410000 Hunan China; 4 Department of Respiratory Medicine, National Key Clinical Specialty, Xiangya Hospital, Central South University, Changsha, 410000 Hunan China

**Keywords:** Lung adenocarcinoma, Immune infiltration, LncRNA, Prognosis

## Abstract

**Background:**

Lung cancer is the most common cancer and cause of cancer‐related mortality worldwide, increasing evidence indicated that there was a significant correlation between tumors and the long non‐coding RNAs (lncRNAs), as well as tumor immune infiltration, but their role in early lung adenocarcinoma (LUAD) are still unclear.

**Methods:**

Gene expression data and corresponding clinical data of early-stage LUAD patients were downloaded from GEO and TCGA databases. 24 kinds of tumor-infiltrating immune cells were analyzed by quantity analysis and univariate cox regression analysis, we divided patients into two subgroups using consensus clustering, recognized the differentially expressed genes (DEGs) in the subgroups, then, established lncRNA risk signature by least absolute shrinkage and selection operator (LASSO) regression.

**Results:**

A total of 718 patients were enrolled in this study, including 246 from GSE31210 dataset, 127 from GSE50081 dataset and 345 from TCGA-LUAD. We identified that Th2 cells, TFH, NK CD56dim cells and Mast cells were prognosis-related(p < 0.05), then established a 5-lncRNA risk signature (risk score = 0.374600616* LINC00857 + 0.173825706* LINC01116 + (− 0.021398903)* DRAIC + (− 0.113658256)* LINC01140 + (− 0.008403702)* XIST), and draw a nomogram showed that the signature had a well prediction accuracy and discrimination.

**Conclusions:**

We identified 4 immune infiltrating cells related to the prognosis of early-stage LUAD, and established a novel 5 immune-related lncRNA signature for predicting patients’ prognosis.

## Background

Lung cancer is the most common malignant neoplasm, which has also the highest morbidity worldwide in recent years [1, 2]. Lung cancer includes two main types: non-small-cell lung cancer (NSCLC, approximately 85%) and small-cell lung cancers (SCLC, approximately 15%) [3], while Lung adenocarcinoma (LUAD) is the most common histological subtypes (about 60%) of NSCLC [4], which has a five-year survival rate of only 22.1% [5].

Tumors grow in a complex network consists of epithelial cells, vascular and lymphatic vessels, cytokines and chemokines, as well as infiltrating immune cells, different types of infiltrating immune cells have different effects on tumor progression [6]. Describing the immune infiltration of the tumor microenvironment can untie the role of immune cells and help establishing a well model for predicting tumor prognosis.

Long noncoding RNA (lncRNA) is a class of RNA with a length of more than 200 nucleotides, which cannot code for proteins. Previous studies showed that lncRNA was mainly involved in the regulation of gene expression at different levels [7], including splicing, transcription, translation, protein modification regulation, etc. it also plays an important role in tumor immunity, such as antigen releasing and presentation, immune cell migration, immune activation, infiltration in tumor tissues, and some other biological processes [8]. Additionally, more and more lncRNAs have been identified act as an oncogene or tumor suppressor in tumor progression, such as HOTAIR in breast cancer [9], MALAT1 in lung cancer [10], and SNHG15 in papillary thyroid carcinoma [11]. Several studies have been undertaken to identify lncRNA-based signature for predicting overall survival for patients with non‐small cell lung cancer (NSCLC) [12, 13].

Until now, the treatment strategy and prognosis of lung cancer are mainly based on the TNM staging system. However, LUAD with the same TNM stage may also have different prognosis due to their different types of pathology and diversity of molecule, the role of molecular markers in the diagnosis, prognosis, and therapy of malignancies is well evidenced [14–18]. However, the roles of immune-related lncRNAs in early-stage LUAD are still unclear. Hence, we sought to quantify infiltrating immune cells in tumors and healthy tissues and identify Immune-related lncRNAs signatures. So, we constructed an immune risk signature that could predict the overall survival (OS) for LUAD patients, the immune risk signature can provide additional prognostic information for the TNM staging system, and we hope it will be of great significance to discover new targeted therapies for early-stage LUAD patients.

## Materials and methods

### Early-stage LUAD patient datasets

**Fig. 1:**
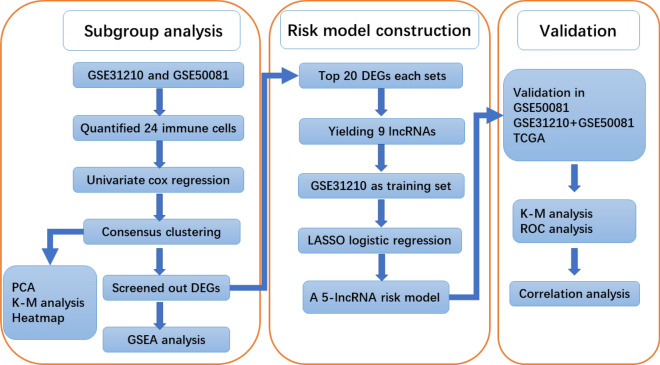
Flowchart of this study. *TCGA* the cancer genome atlas, *GSEA* gene set enrichment analysis, *PCA* principal component analysis, *K–M analysis* Kaplan–Meier analysis, *ROC* receiver operating characteristic

Figure 1 showed a flowchart of the steps involved in this study. Gene expression data and corresponding clinical data of LUAD patients were downloaded from the Gene Expression Omnibus (GEO) databases and the cancer genome atlas (TCGA) databases. According to the inclusion criteria of early-stage (stage I and II) LUAD patients. The gene expression data and clinical data of LUAD patients were downloaded from TCGA database by “TCGAbiolinks” package [19] in R (4.0.2). A total of 718 patients were enrolled in our study, including 20 normal samples and 226 patients from GSE31210 dataset, 127 patients from GSE50081 dataset and 345 patients from TCGA-LUAD. Of these, the patients from GSE31210 dataset were used as a training cohort to build a risk signature, while the other two datasets (GSE50081 and TCGA-LUAD) were used as the verifying cohort to validate the signature.

### Acquisition and processing of lncRNA expression profile

We downloaded the data having been processed by the Robust Multichip Average (RMA) algorithm. lncRNA expression data of LUAD patients were obtained by performing a probe reannotation pipeline as previous described [20, 21]. “Homo_sapiens.GRCh38.90.chr.gtf” was used to annotate the probe, and gene biotype was not protein_coding but transcript_biotype was one of the following: “sense_overlapping”, “lincRNA”, “3prime_overlapping_ncRNA”, “processed_transcript”, “sense_intronic”, “bidirectional_promoter_lncRNA”, “non_coding” was considered to be a lncRNA. The average expression value was used when a gene was mapped to multiple probes. As for TCGA data, we downloaded the RNA sequencing expression profiles with the type of counts from the TCGA (https://portal.gdc.cancer.gov), then we extracted lncRNAs using the previous method.

### Quantification of infiltrating immune cells

In terms of quantifying immune infiltrating cells, a review [22] published in *Cancer Immunology* in 2018 summarized several methods currently used, we used the ssGSEA (single-sample geneset enrichment analysis) as it described to quantify infiltrating immune cells, one of the biggest advantages of this method is that we can customize and quantify the types of immune infiltrating cells by using the "GSVA" package (R package). With the widely recognized and used immune cell marker genes provided by Bindea et al. [23], we used these marker genes to extract 24 immune cells’ information. Therefore, based on the gene expression data and marker genes, we can use the ssGSEA to quantify infiltrating immune cells.

### Differences and correlation in tumor and normal tissues

To compare the differences of immune cells between LUAD and normal tissue, The “pheatmap” R package and the “vioplot” R package were used for drawing the plots, and we also used the “corrplot” package to show the correlation of immune cells in tumor tissues.

### Identification of immune cells and subgroup analysis

To determine the prognostic value of immune cells, we performed univariate Cox regression analysis on the 24 immune cells in GSE31210 by using the “survival” package in R (v4.0.2) and measured the hazard ratios (HRs) with 95% confidence intervals (CIs),it indicated that immune cells were correlated with overall survival (OS) and considered prognostic immune cells with the p-value < 0.05, immune cells with HRs < 1 were considered to be risk factors, while HRs > 1 were protective factors, and further verify with GSE50081. We selected the immune cells that play the same role in the tumor prognosis of the two data sets, and the amount of corresponding immune infiltration cells was used to cluster the LUCD patients into different subgroups with the use the “ConsensusClusterPlus” package. We analyzed the two subgroups using principal component analysis (PCA) with the R package in R (v4.0.2) to study the immune cells in different subgroups to judge whether our cluster was correct, and a heatmap was used to show the 24 immune cells in subgroups of the dataset.

### Identification of DEGs

Use the “limma” package to identify the difference between the subgroups of GSE31210 and GSE50081 datasets, that is, to screen out the DEGs (differentially expressed genes) between the immune subgroup with good prognosis and that with poor prognosis. The screening condition were p-value < 0.05 and |logFC|≥ 1, to pursue their possible functions, we performed GSEA (gene set enrichment analysis) [24] by the “clusterProfiler” R package, and it was considered a statistically significant difference with the p-value < 0.05.

In addition, in order to determine the prognostic value of lncRNA, lncRNA was extracted from DEGs, and logFC ± top20 were taken from the two data sets respectively, a total of 40 genes (p < 0.05), the intersection was taken, yielding 9 genes.

### Identification of hub-genes and risk-score model construction

Hub-genes were several lncRNAs those closely related to the prognosis of LUAD patients. We proposed GSE31210 as the experimental cohort, and the data of GSE50081 and TCGA were used for validation. Firstly, we combined their gene expression matrices and removed the inter-batch difference using the combat function of “sva”package, resulting in a total of 698 samples, and we’ve already identified 9 lncRNAs (associated with immunity) through the previous method, next, we did least absolute shrinkage and selection operator (LASSO) logistic regression [25] in 226 samples of GSE31210 to screen out hub-genes and determine their coefficients for developing the risk score model.

We constructed Kaplan–Meier survival curve of LUCD patients and divided the patients into high-risk group and low-risk group according to the cutoff found by the Survminer R package, and the receiver operating characteristic (ROC) and the area under the curve (AUC) were used to assess the prognostic performance of our risk score model.

### Building and validation of a nomogram

A nomogram is a simple graph which used to predict cancer prognosis, we used the previously identified hub genes to construct a nomogram by the “rms” package, then, we draw a calibration plot to verify the nomogram, we calculated the concordance index (C-index) and plotted ROC curves were used to evaluate the predictive ability of the nomogram to measure the nomogram’s veracity.

## Results

### Identification of infiltrating immune cells and survival analysis

**Fig. 2:**
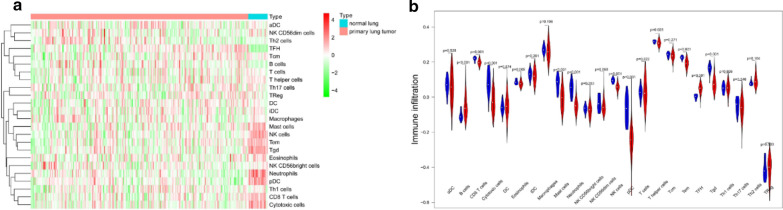
The amount of 24 immune cells between normal and tumor tissues. **a** Heatmap; **b** vioplot

**Fig. 3:**
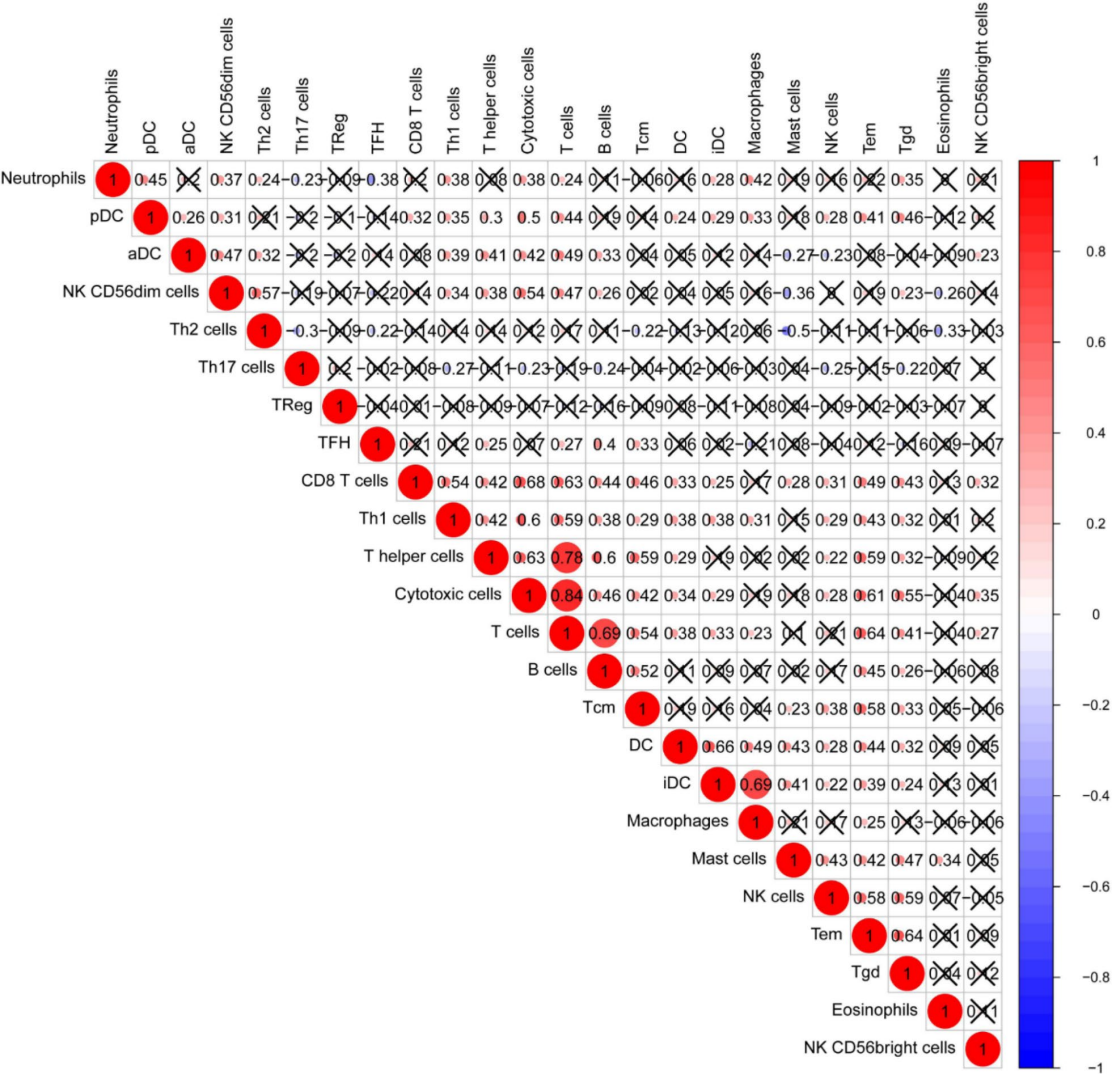
Spearman correlation of immune cells in tumor tissues

After the quantification of infiltrating immune cells, GSE31210 was used as the training dataset, we plotted a heatmap and a vioplot to show the amount of 24 immune cells between normal and tumor tissues, the results were shown in Fig 2a, b, 12 kinds of immune cells (B cells, CD8 T cells, Cytotoxic cells, Eosinophils, Mast cells, Neutrophils, NK cells, pDC, T helper cells, Tem, TFH and Tgd) were differentially expressed between them (p < 0.05).And the correlation of immune cells in tumor tissues was shown as Fig. 3, the amount of Th2 cells and Mast cells were negatively correlated with each other in LUAD, while the amount of Th2 cells and NK CD56dim cells were positively correlated.

**Fig. 4:**
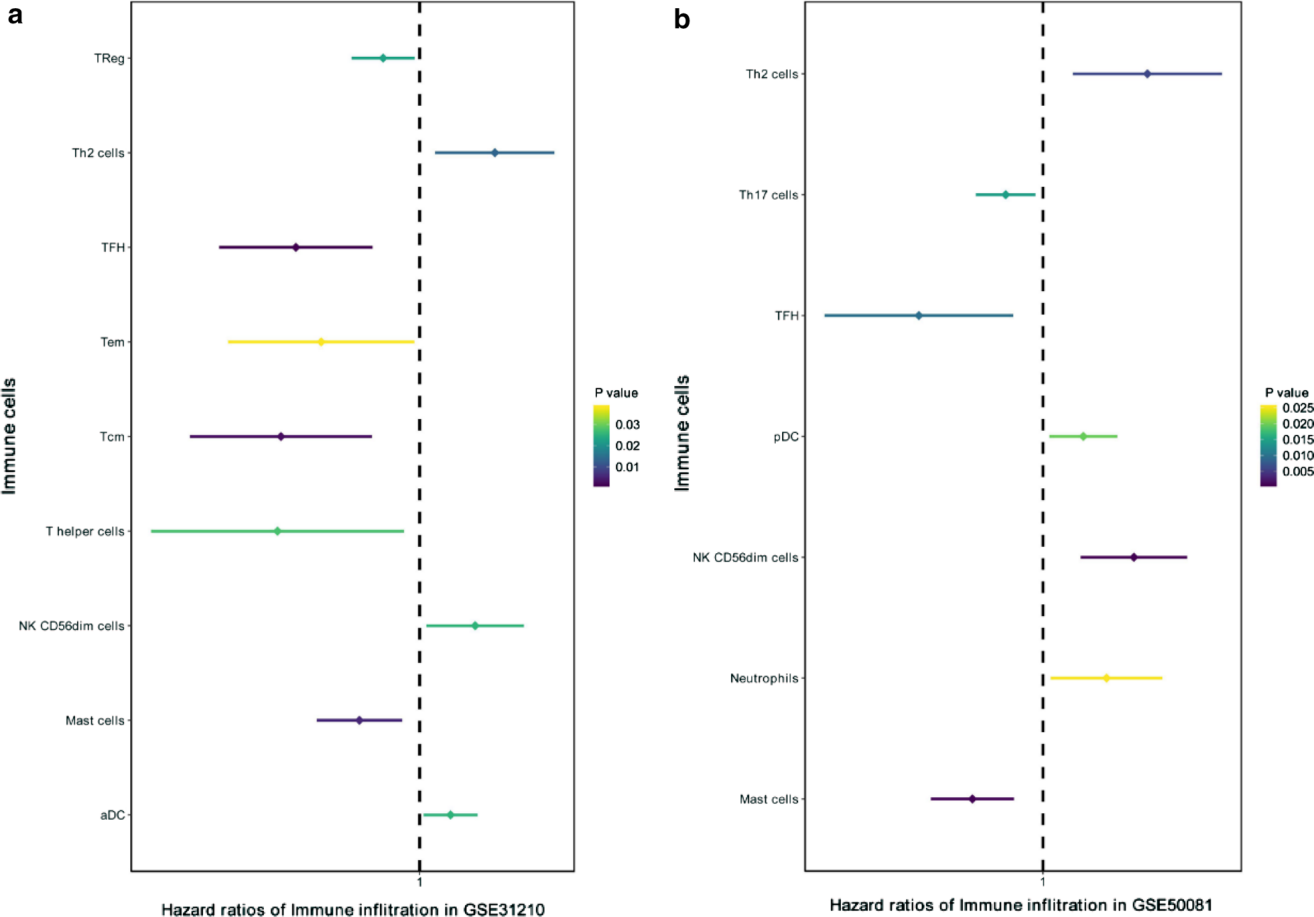
Forest map of univariate Cox analysis in LUAD. **a** GSE31210 dataset; **b** GSE50081 dataset. *LUAD* lung adenocarcinoma

We performed univariate Cox regression analyses for the expression of 24 immune cells in GSE31210, and further verified with GSE50081. The results were shown in Fig. 4: Th2 cells, TFH, NK CD56dim cells, and Mast cells play the same role in the tumor prognosis of the two datasets, Th2 cells and NK CD56dim cells are protective factors, while TFH and Mast cells are the opposite.

### Analysis of subgroup

**Fig. 5:**
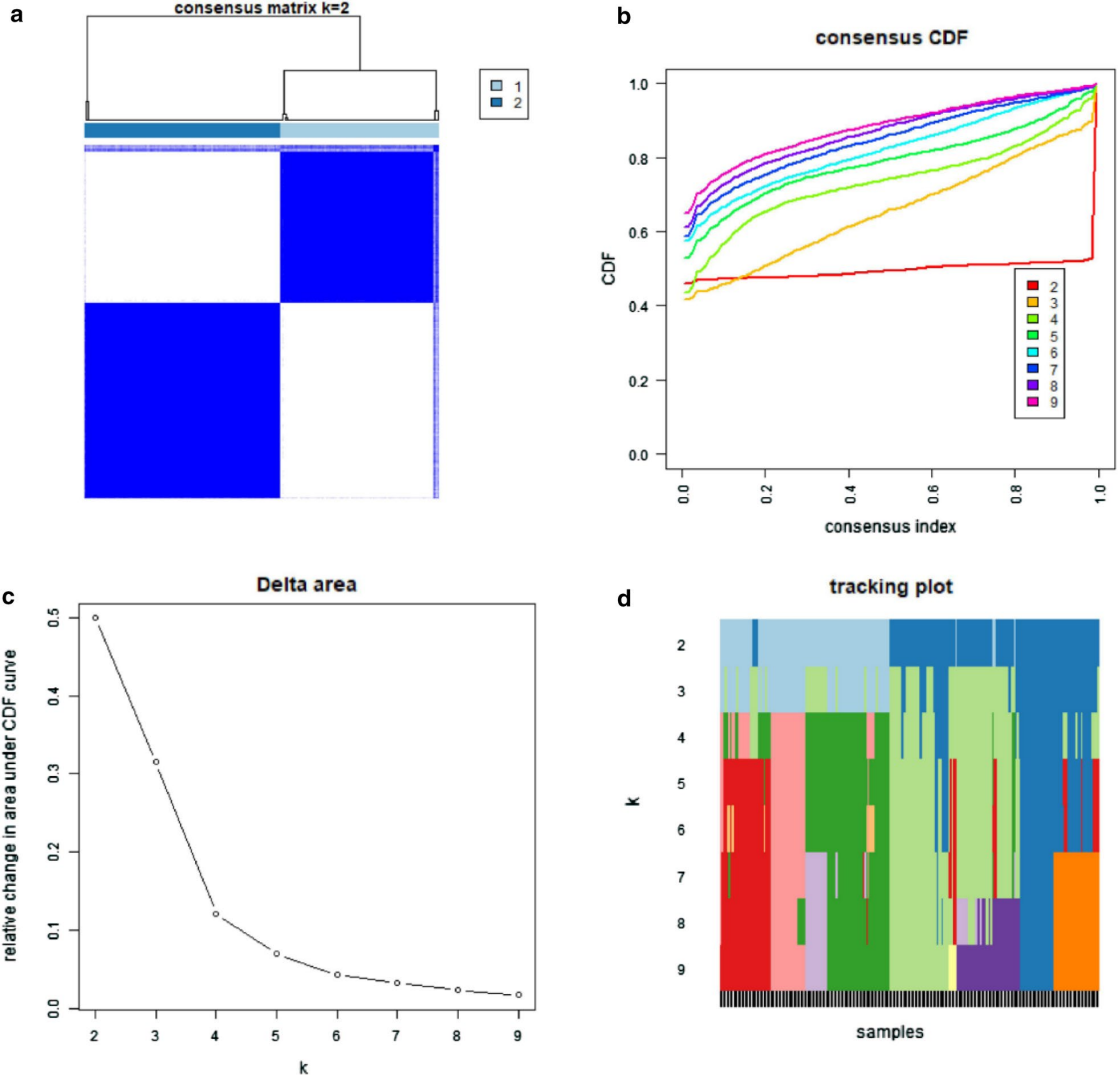
Identification of consensus clusters by immune cells in GSE31210. **a** Consensus clustering matrix for k = 2; **b** consensus clustering cumulative distribution function (CDF) for k = 2–9; **c** relative change in area under CDF curve fork = 2–9; **d** the tracking plot for k = 2–9

**Fig. 6:**
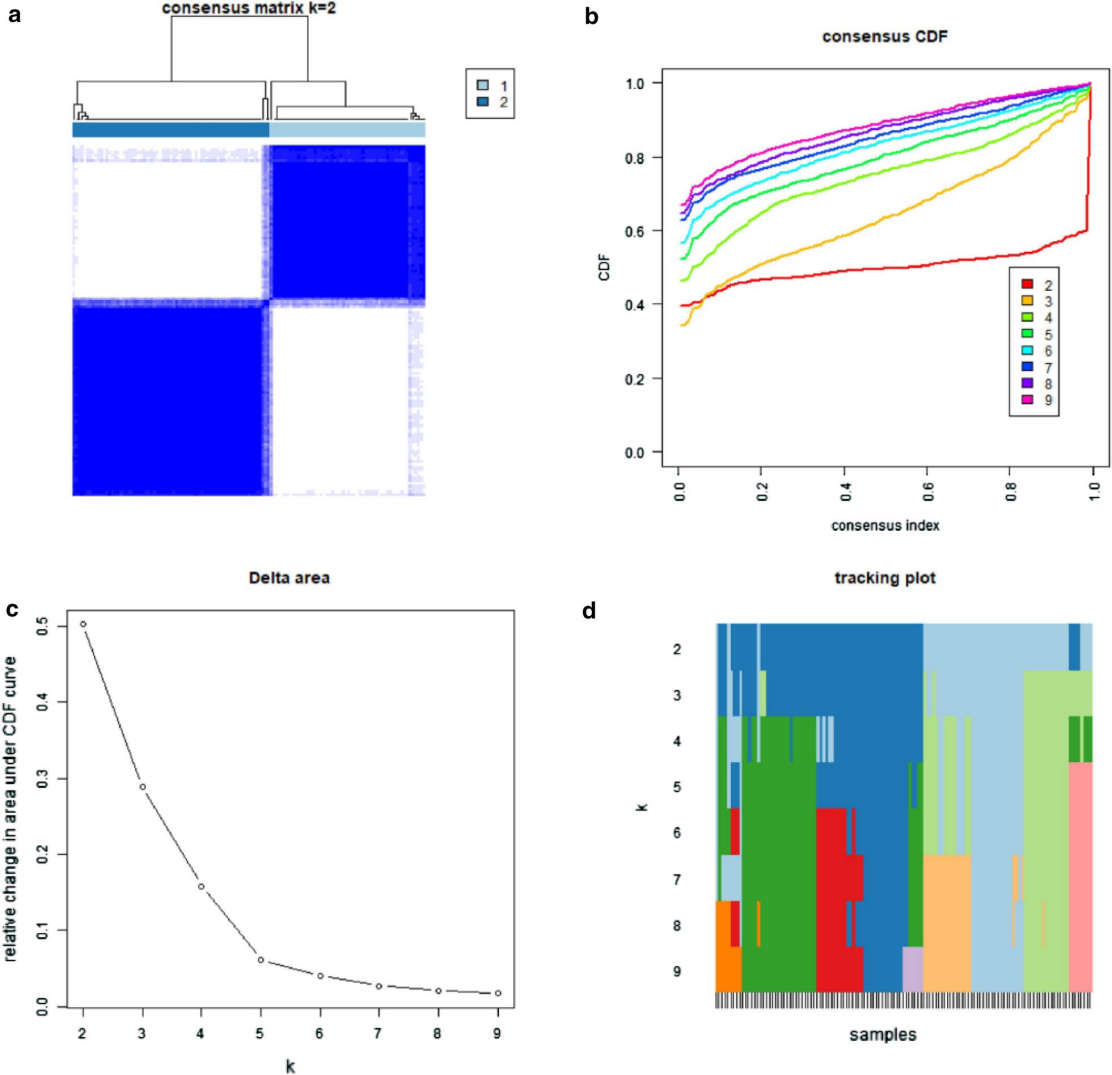
Identification of consensus clusters by immune cells in GSE50081. **a** Consensus clustering matrix for k = 2; **b** consensus clustering cumulative distribution function (CDF) for k = 2–9; **c** relative change in area under CDF curve for k = 2–9; **d** the tracking plot for k = 2–9

**Fig. 7:**
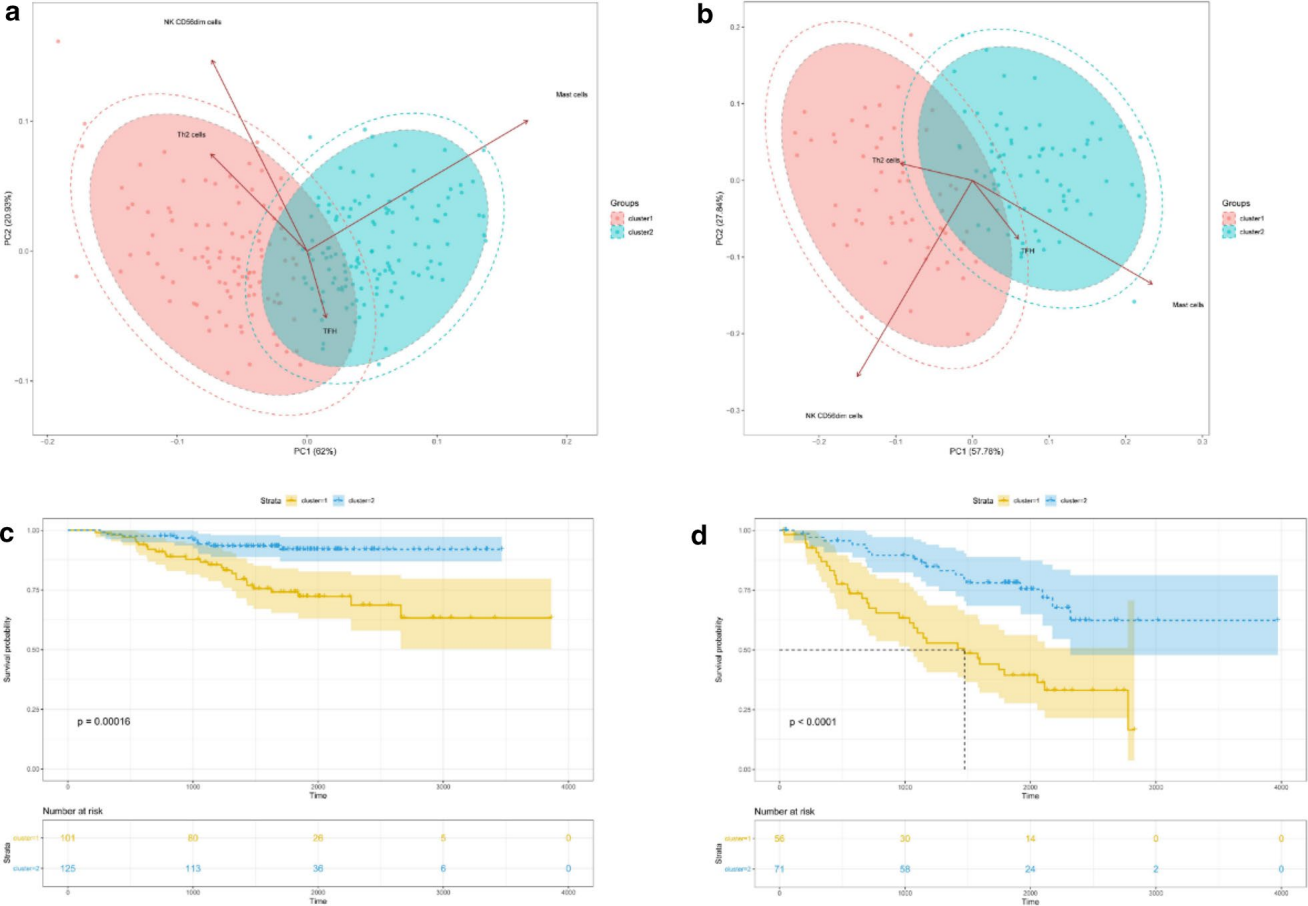
Principal component analysis (PCA) of 4 immune cells and Kaplan–Meier overall survival (OS) curve for patients of two clusters of the GEO datasets. **a** PCA in GSE31210; **b** PCA in GSE50081; **c** Kaplan–Meier overall survival (OS) curve for patients for 226 LUAD patients (cluster1: n = 101, cluster2: n = 125) in GSE31210; **d** Kaplan–Meier overall survival (OS) curve for patients for 127 LUAD patients (cluster1: n = 56, cluster2: n = 71) in GSE50081

**Fig. 8:**
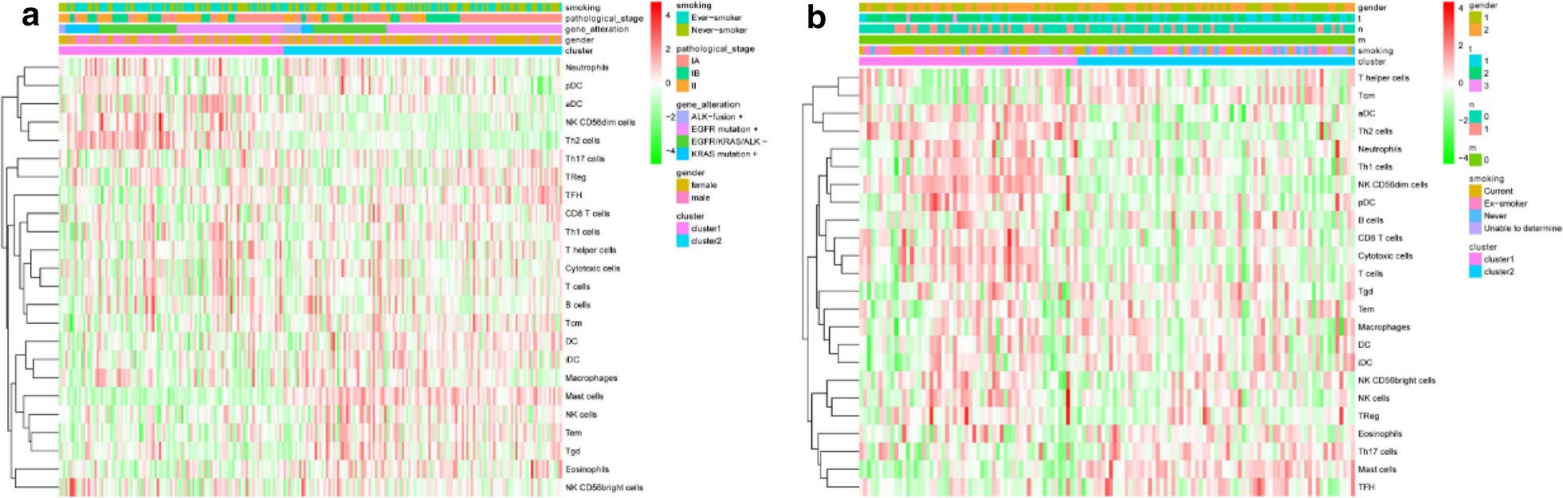
Heatmap and clinicopathologic features of the two clusters (cluster1, 2). **a** Heatmap in GSE31210; **b** heatmap in GSE50081

According to the amount of the 4 immune cells (Th2 cells, TFH,NK CD56dim cells and Mast cells) identified above, k = 4 seemed to be the optimal choice with clustering stability increasing from k = 2—9 (Fig. 5b–d) in GSE31210, while k = 5 in GSE50081(Fig. 6b–d). However, we found that only when k = 2, the interference between subgroups was minimal in GSE31210 and GSE50081 (Figs. 5a, 6a). Therefore, the datasets were divided into two subgroups (cluster1&2), we also analyzed the immune cells in two subgroups by principal component analysis (PCA), it showed a clear distinction between them (Fig. 7a, b), which further indicated our classification was meaningful. In addition, we found that the cluster1 had a significantly shorter overall survival (OS) than those in cluster2 (Fig. 7c, d). And the heatmap (Fig. 8a, b) was plotted to show the amount of 24 immune cells and the distribution of clinicopathologic features between cluster1 and cluster2.

### Identification of DEGs and construction of risk score model

**Fig. 9:**
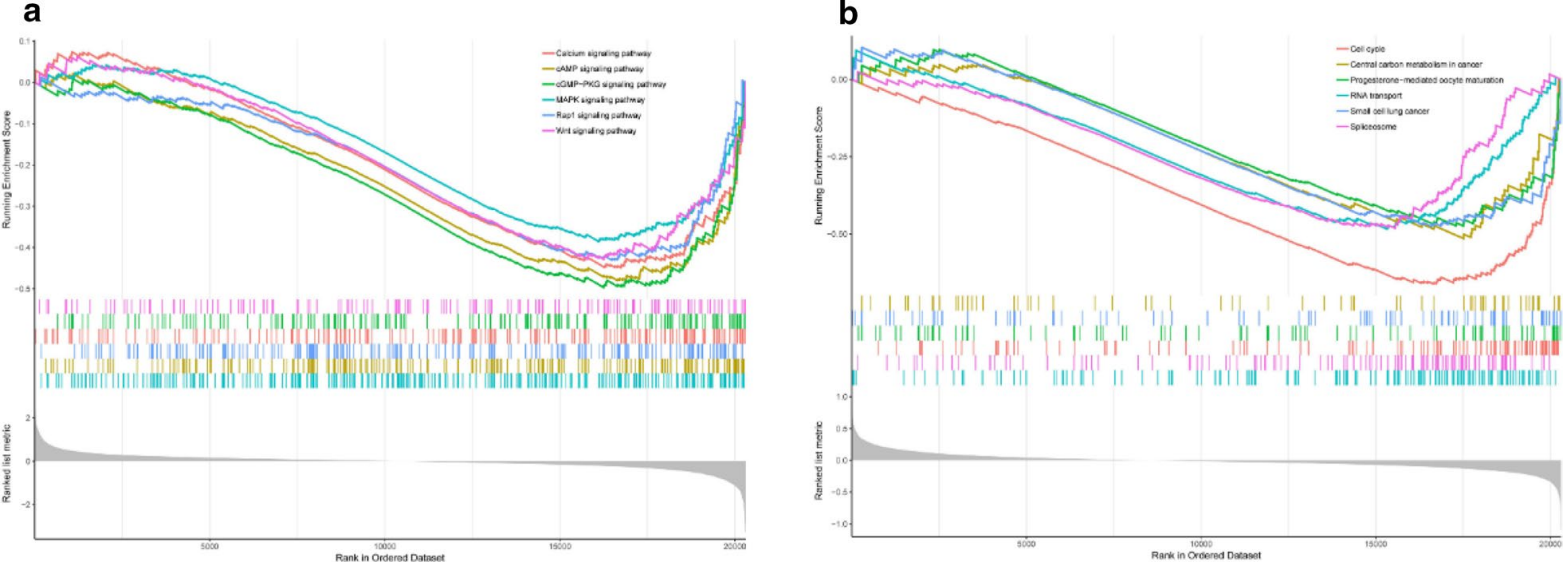
Gene set enrichment analysis (GSEA). **a** the top 6 enriched pathways in GSE31210; **b** the top 6 enriched pathways in GSE50081

We screened out differentially expressed genes (DEGs) (lncRNA) between cluster1 and cluster2 in GSE31210 and GSE50081, then, we performed GSEA (gene set enrichment analysis) to seek out the possible main functional pathways involved, respectively. As a result of 74 enriched pathways involved in GSE31210 and 49 pathways in GSE50081, the top 6 enriched pathways in GSE31210 were Calcium signaling pathway, cAMP signaling pathway, cGMP-PKG signaling pathway, MAPK signaling pathway, Rap1 signaling pathway and Wnt signaling pathway (Fig. 9a), while the top 6 enriched pathways in GSE50081 were Cell cycle, Central carbon metabolism in cancer, Progesterone-madiated oocyte maturation, RNA tansport, Small cell lung cancer and Spliceosome (Fig. 9b), and there were 22 pathways involved in both datasets (Table 1), the above results suggested that the two clusters determined by consensus clustering had different clinical characteristics and prognosis of LUAD. In these two datasets, the main functional pathways seemed to be different, but they also had many of the same pathways (Table 1), it showed that two datasets were more likely to be associated with the malignancy of LUAD. These results indicated that the occurrence, development and prognosis of LUAD involved multiple pathways.

**Table 1:** Pathways involved in both datasets (GSE31210 and GSE50081) of Gene Set Enrichment Analysis (GSEA)

No	ID	Description
1	hsa05144	Malaria
2	hsa00982	Drug metabolism—cytochrome P450
3	hsa04610	Complement and coagulation cascades
4	hsa04726	Serotonergic synapse
5	hsa04014	Ras signaling pathway
6	hsa05206	MicroRNAs in cancer
7	hsa04218	Cellular senescence
8	hsa01200	Carbon metabolism
9	hsa04114	Oocyte meiosis
10	hsa04914	Progesterone-mediated oocyte maturation
11	hsa03013	RNA transport
12	hsa03440	Homologous recombination
13	hsa03460	Fanconi anemia pathway
14	hsa01230	Biosynthesis of amino acids
15	hsa04115	p53 signaling pathway
16	hsa00051	Fructose and mannose metabolism
17	hsa00240	Pyrimidine metabolism
18	hsa03008	Ribosome biogenesis in eukaryotes
19	hsa04110	Cell cycle
20	hsa03430	Mismatch repair
21	hsa03410	Base excision repair
22	hsa03030	DNA replication

### Identification of hub genes and construction of risk score model

**Fig. 10:**
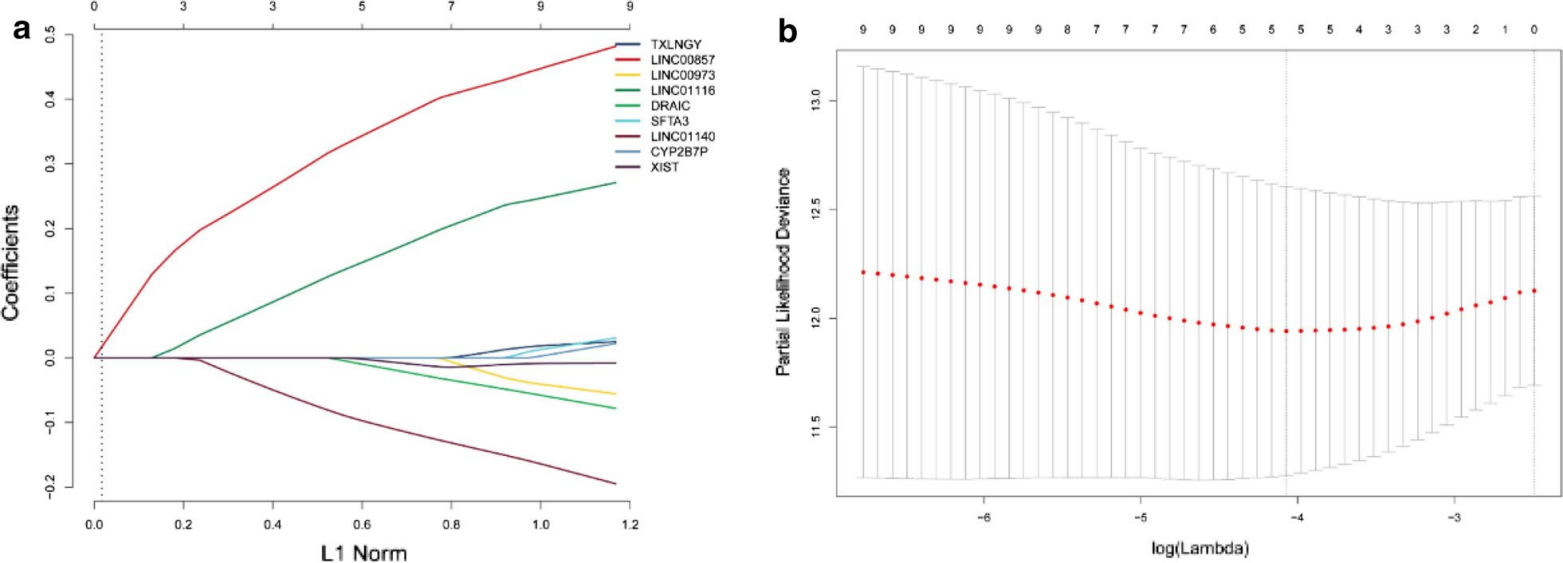
**a** Distribution of the LASSO coefficients for 9 lncRNAs; **b** partial likelihood deviation of the LASSO coefficient distribution. Vertical dashed lines indicate lambda

We screened out 9 lncRNAs (associated with immunity) using the method described in methods, they were"TXLNGY","LINC00857","LINC00973","LINC01116","DRAIC","SFTA3","LINC01140","CYP2B7P" and "XIST". We proposed GSE31210 dataset as the experimental cohort, and the data of GSE50081 and TCGA were used for validation, for further screening out the lncRNA related to prognosis and developing a risk score model, we conducted LASSO logistic regression analysis to the 9 lncRNAs in 226 samples of GSE31210.The LASSO results (Fig. 10a, b) showed that 5 lncRNAs (hub genes) were the powerful prognostic factors, and then constructed the risk score model as: risk score = 0.374600616 * LINC00857 + 0.173825706 * LINC01116 + (− 0.021398903) * DRAIC + (− 0.113658256) *  LINC01140 + (− 0.008403702) * XIST.

**Fig. 11:**
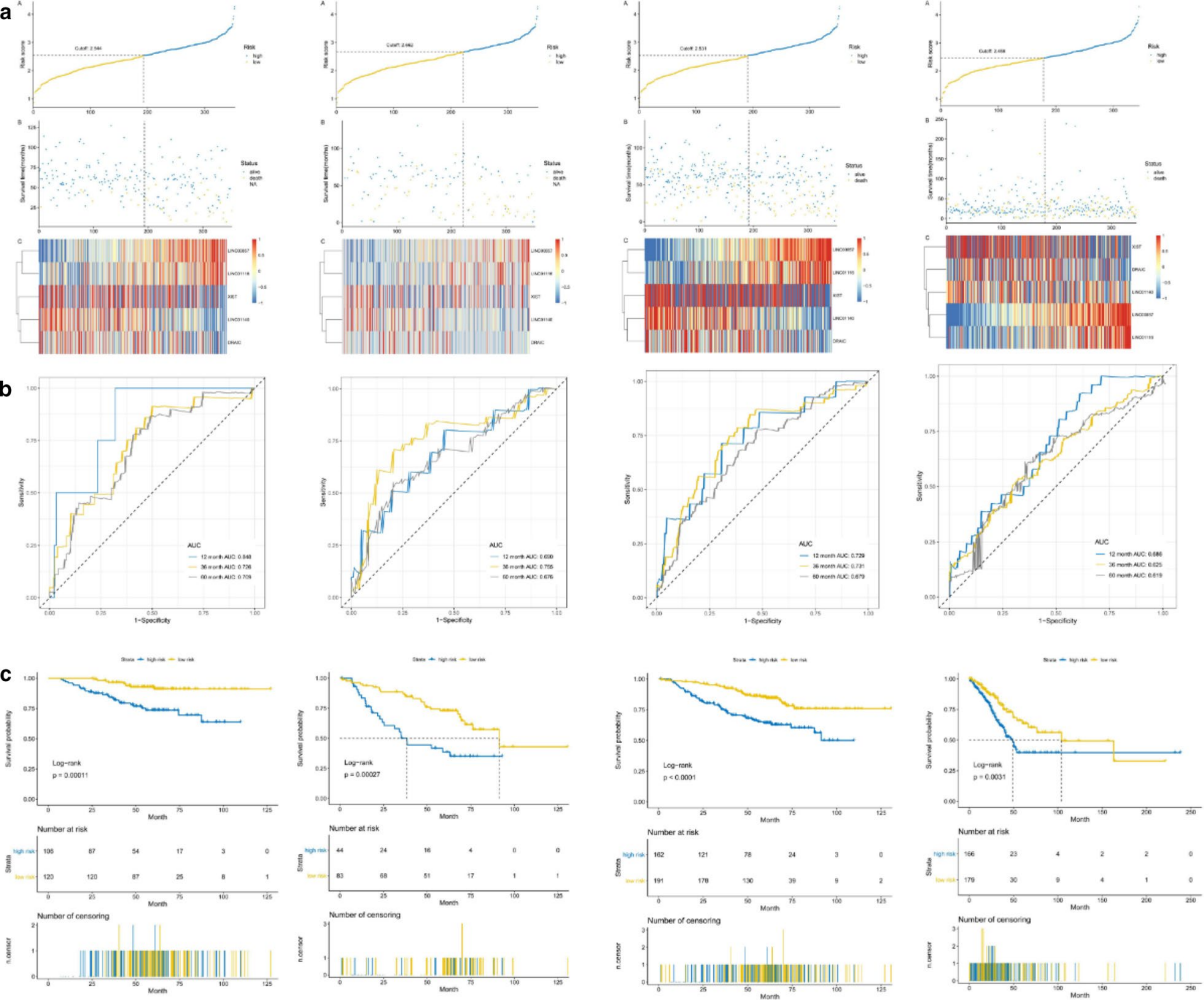
Risk score, heatmap of lncRNA, time-dependent ROC analysis, and Kaplan–Meier curve of the 5-lncRNA signature in indifferent datasets. **a** Risk score, heatmap of lncRNA; **b** Time-dependent ROC analysis; **c** Kaplan–Meier curve. It was GSE31210 set, GSE50081 set, GSE31210 + GSE50081 set and TCGA set from the left to right. *AUC* area under the ROC curves, *ROC* receiver operating characteristic, *TCGA* The Cancer Genome Atlas

We constructed Kaplan–Meier survival curve of early-stage LUCD patients which divided the patients into high-risk and low-risk group according to the optimal cutoff, and the area under the curve (AUC) of the subject's work characteristics (ROC) was used to assess the clinical prognostic ability of this risk score model, therefore, we plotted the risk score distribution, the time-dependent ROC curve and the survival analysis of the training set (GSE31210), validating set (GSE50081 set, GSE31210 + GSE50081 set and TCGA set) (Fig. 11). The area under the ROC curves (AUCs) of the OS prognostic model were as follows: 12 month AUC: 0.848, 36 month AUC: 0.726, 60 month AUC: 0.709; 12 month AUC: 0. 690, 36 month AUC: 0.755, 60 month AUC: 0.764; 12 month AUC: 0.729, 36 month AUC: 0.731, 60 month AUC: 0.679; 12 month AUC: 0.686, 36 month AUC: 0.625, 60 month AUC: 0.619. In conclusion, it suggested well-prediction performance of the 5-lncRNA signature for survival prediction.

### Building a nomogram based on hub genes

**Fig. 12:**
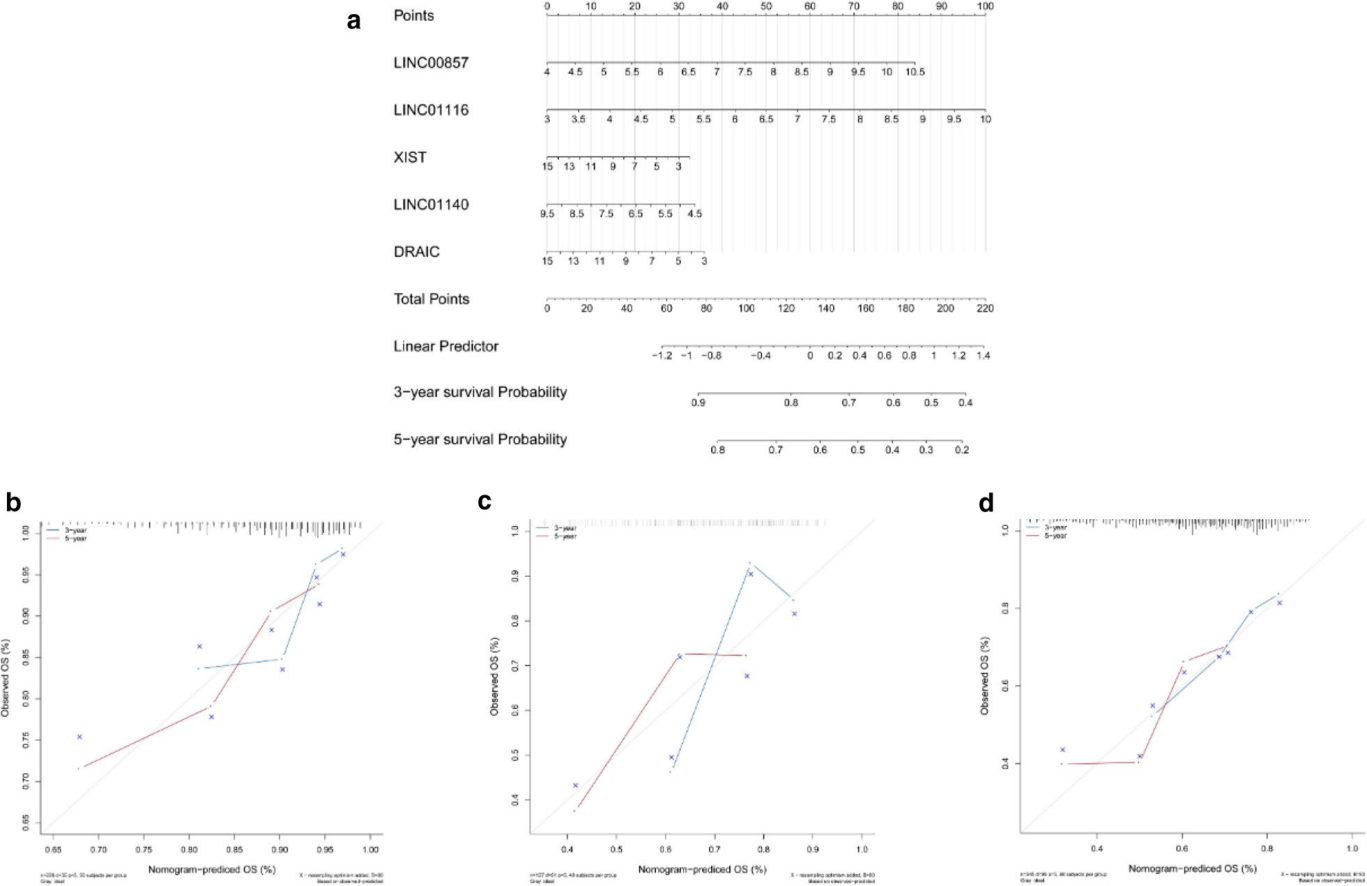
**a** The nomogram-predicted OS for LUAD patients; **b**–**d** the calibration curves of GSE31210 dataset, GSE50081 dataset and TCGA dataset. The 3-y survival probability curve is the blue line, the 5-y survival probability curve is the red line, and the ideal curve is gray. LUAD, lung adenocarcinoma; OS, overall survival; TCGA, The Cancer Genome Atlas

We built a nomogram to predict of 3-year and 5-year OS of LUAD patients (Fig. 12a). Calibration curves and the C-index were used to assess the discrimination and accuracy of the nomogram, the C-index was 0.71121105 in GSE31210 dataset, the C-index was 0.65793781 in GSE50081 dataset, and 0.63457270 in TCGA dataset, the calibration curves for the 3-year and 5-year survivals showed good agreement to the ideal curves in both training set and validation set (Fig. 12b–d), indicating good prediction performance of the nomogram.

### Correlation analysis between hub-genes and infiltrating immune cells

**Fig. 13:**
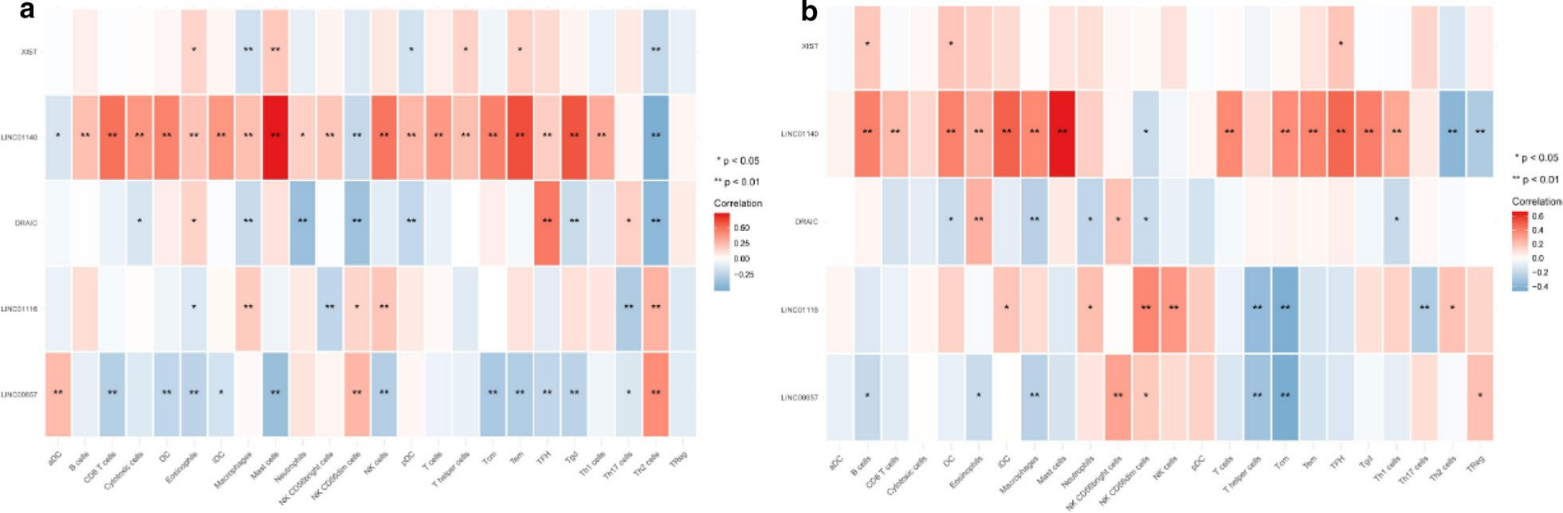
The correlation between 5 hub genes and infiltrating immune cells. **a** GSE31210 dataset; **b** GSE50081 dataset

To further verify the correlation between hub-genes and infiltrating immune cells, we performed Spearman correlation analysis using the “ggstatsplot” package, the result showed a significant correlation between them in both GSE31210 dataset and GSE50081 dataset (Fig. 13).

## Discussion

In this study, we identified immune cells and lncRNAs that were strongly correlated with the prognosis of early-stage LUAD patients, thereby deepening the understanding of the immune mechanism of early-stage LUAD. Firstly, we use single-sample Gene Set Enrichment Analysis (ssGSEA) to quantify tumor immune infiltrating cells. Quantitative analysis and univariate COX regression identified 4 immune cells that played the same role in the prognosis of tumors in the two sets. According to the level of immune cells, through consensus clustering, the two sets were divided into two immune subgroups (Cluster1/2) with good or poor prognosis, Cluster1/2 group not only affects the prognosis, but also related to the key signal pathways of LUAD. In addition, according to the differentially expressed genes (DEGs) in the subgroups, on the one hand, GSEA was used to find the enriched pathways that ranked high, and on the other hand, hub genes closely related to prognosis were screened by LASSO regression and a 5-lncRNAs risk score model was successfully established, and patients were divided into high-risk and low-risk group, the Kaplan–Meier overall survival (OS) curve showed that patients in the low-risk group have a longer OS than those in high-risk group. At the same time, the AUC also showed that the signature had good prognostic value regardless of whether they were in the experimental cohort or the verification cohort, finally, the nomogram also showed a well prediction performance, it further proved that these lncRNAs could potentially serve as biomarkers for the prognosis of early-stage LUAD patients.

Our study showed there were 4 immune cells played vital roles in early-stage LUAD. Among the 4 identified prognostic immune cells in our study, Th2 cells and NK CD56dim cells are protective factors, in contrast to TFH and Mast cells. Lorvik et al. [26] have reported that Th2 cells and DPT cells interact each other to facilitate the escape of urological cancer, while it reported that Th2 cells were protective factors in gastric tumor [27], these suggest that TH2 cells may play different roles in diverse tumors. A previous study indicated that IL-15–primed NK cel ls could survive in the ROS-rich tumor microenvironment, which is conducive to those smokers with lung cancer [28]. A Randomized Phase II Clinical Trial showed TKD/IL2-activated-NK cells were beneficial to advanced NSCLC patients after radiochemotherapy [29]. Zhenxing Guo et al. also showed that circulating Tfh (cTfh) was of great importance for the pathogenesis of NSCLC patients [30]. It reported that Mast cells (MC) was involved in the regulation of innate and adaptive immune responses [31], and a growing number of studies show that mast cells play a vital role in antineoplastic immunity in recent years [32, 33].

In the past, when exploring cancer-related lncRNAs, most studies only focused on the prognosis and predictive ability of a single molecule, but the process of tumor development and metastasis is often the result of a synergistic effect of multiple molecules. We identified 5 hub genes (LINC00857, LINC01116, DRAIC, LINC01140 and XIST) signature performing a good prediction in early-stage LUAD patients. To date, more and more lncRNAs have been discovered and some of them are well characterized in various cancers. But the 5 lncRNAs remain uncharacterized in early-stage LUAD, while they have been reported to varying degrees in lung cancer or other diseases by previous studies. A study [34] showed that LINC00857 promoted the progression of LUAD, it regulated the cell proliferation, glycolysis and apoptosis of LUAD cells by targeting miR-1179/SPAG5 axis. Another study showed that LINC00857 regulated the progression of lung cancer through cell cycle regulation [35], there was also a study indicated that cell cycle, Hippo, TGFβ, and p53 pathway alterations were associated with poor disease-free survival (DFS) in early-stage LUAD [36], these results consistent with our results that cell cycle and p53 signaling pathway were the predicted pathways. LINC01116 was overexpressed in various tumors, such as osteosarcoma, nasopharyngeal carcinoma and some others [37–39], another study found that LINC01116 was overexpressed in LUAD tissues and cell lines and suggested that LINC01116 may act as an oncogene in LUAD [40]. And LINC01116 also contributed to gefitinib resistance of NSCLC cells [41]. Marina et al. demonstrated that LINC01140 was an important regulator of proliferation and inflammation in human lung fibroblasts [42]. LINC01140 may be a potential biomarker for the prognosis of patients with breast cancer [43] and gastric cancer [44]. However, there has no study show the connection between LINC01140 and LUAD. XIST was associated with chemotherapy resistance in non-small cell lung cancer (NSCLC) [45], and it also can expedite the progression of LUAD [46]. As for DRAIC, it was reported inhibiting prostate cancer progression through suppression of NF-κB activation.

The above 5 lncRNAs have been reported to varying degrees in lung cancer or other diseases. And this is the first time that we have linked lncRNAs with immune infiltration to predict the prognosis of early-stage LUAD. Through our research results, we can see that DEGs affect the prognosis of early-stage LUAD may involve many pathways, and it highlighted that lncRNA may serve as prognostic molecular markers and therapeutic target for patients with early-stage LUAD, and it requires further research to explore the specific mechanisms.

## Conclusion

In this study, we identified 4 kinds of immune infiltrating cells related to the prognosis of early-stage LUAD, and also successfully established a novel 5 immune-related lncRNA signature for predicting patients prognosis with early-stage LUAD, these results may provide new ideas of molecular mechanism for future research and relevant target therapy of early-stage LUAD.

## Data Availability

The datasets supporting the conclusions of this article are available in the Gene Expression Omnibus (GEO) database and the cancer genome atlas (TCGA) database.
